# Supplementary material for: Identification of photoperiod-induced specific miRNAs in the adrenal glands of Sunite sheep (Ovis aries)
Source: Front Vet Sci. 2022 Jul 22;9:888207. doi: 10.3389/fvets.2022.888207 (PMC9354845; doi:10.3389/fvets.2022.888207)
Supplement: Supplementary file 1 [file Data_Sheet_1.ZIP › Supplementary file/miRDeep2 file.zip!/miRDeep2 file/Novel_Structure/SP42A2/Novel_706.pdf]

Provisional ID	: NC_019480.2_38599
Score total	: 0.3
Score for star read(s)	: -1.3
Score for read counts	: 0
Score for mfe	: 0
Score for randfold	: 1.6
Score for cons. seed	:
Total read count	: 78
Mature read count	: 70
Loop read count	: 0
Star read count	: 8

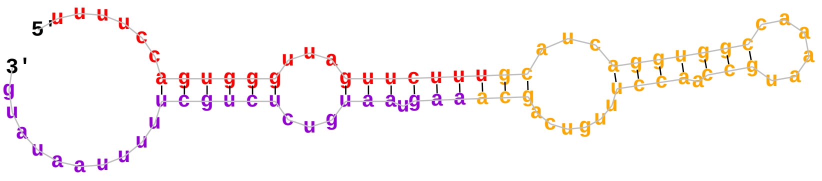

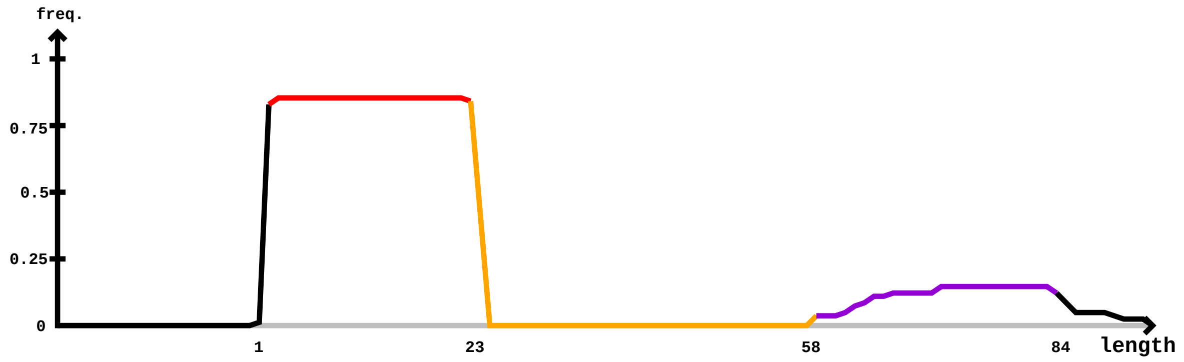


```
                   Mature                                                   Star
5'- ucuuuccuaguaucagggucuuuuccaguggguuaguucuuugcaucagguggccaaaaugccaaccuuugucagcaaaguaaugucucugcuuuuuaauaugcugucuagg  -3'   obs
    ucuuuccuaguaucagggucuuuuccaguggguuaguucuuugcaucagguggccaaaaugccaaccuuugucagcaaaguaaugucucugcuuuuuaauaugcugucuagg        exp
    .....(((((...(((.((.((....((((((...(((((((((...(((((((......))).))))......)))))).)))...))))))....)).)).))).)))))   reads   mm   sample
    ...................cuuuuccagugAguuaguucuu.......................................................................   1       1    seq
    ....................uuuuccagugAguuaguucuuu......................................................................   63      1    seq
    ....................uuuuccaguggguuaguucuuu......................................................................   1       0    seq
    ....................uuuuccagugAguuaguucuuug.....................................................................   3       1    seq
    .....................uuuccagugAguuaguucuuu......................................................................   2       1    seq
    .............................................................................aaguaaugucucugcuuuuuaauau..........   1       0    seq
    .............................................................................aaCuaaugucucugcuuuuuaauaug.........   1       1    seq
    .............................................................................aaguaaugucucugcuuuuuaauaug.........   1       0    seq
    ................................................................................uaaugucucugcuuuuuaauaug.........   1       0    seq
    .................................................................................aaugucucugcuuuuuaauaugc........   1       0    seq
    .................................................................................aaugucucugcuuuuGaauaugcugucu...   1       1    seq
    ..................................................................................augucucugcuuuuuaauau..........   1       0    seq
    ...................................................................................ugucucugcuuuuuaauauA.........   1       1    seq
    ...................................................................................ugucucugcuuuuCaauaugc........   1       1    seq
    .....................................................................................ucucugcuuuuuaauaugcuguU....   1       1    seq
    ..........................................................................................gcuuuuuaauaugcugucuagg   1       0    seq
    ...........................................................................................Ucuuuuuaauaugcugucuagg   1       1    seq
```
